# Pain perception among patients with fixed appliances over invisalign aligners

**DOI:** 10.6026/973206300210301

**Published:** 2025-03-31

**Authors:** Kolasani Srinivasa Rao, B. Rama Mohan Reddy, M. Kaladhar Naik, Gnan Prakash Dakarapu, Mate Shreanshi Anil, Venugopal G.M

**Affiliations:** 1 Department of Orthodontics and Dentofacial Orthopaedics, Government Dental College and Hospital, Vijayawada 520004 andhra Pradesh, India; 2 Department of Orthodontics and Dentofacial Orthopaedics, Government Dental College, Kadapa andhra Pradesh, India

**Keywords:** Invisalign® aligners, fixed appliance, pain perception, treatment

## Abstract

The pain perception among patients treated with fixed appliances over Invisalign aligners is of interest. Hence, a total of 75 participants were divided equally into 3 groups namely Group I - Invisalign, Group II - self ligating fixed appliances and Group III - conventional fixed appliance treatment. Perception of pain among patients was evaluated using a Visual Analog Scale (VAS) at 6 hours, 24 hours, 48 hours and 1 week. Patients treated with Invisalign aligners showed lower pain compared to fixed appliances.

## Background:

Over time, more and more people are getting orthodontic treatment and one of their main concerns is the pain and discomfort that comes with it [1]. According to earlier research, between 91 and 95 percent of study participants report feeling of some level of pain [2, 3]. Prior to the start of orthodontic treatment, patients also cited pain as a major source of anxiety and dread [1]. Self-ligating brackets were found to cause much less pain during orthodontic treatment than conventional brackets, according to Tecco *et al.* [4]. Following their adjustment visit, patients in the fixed appliance group showed a statistically higher intake of pain medication than those in the removable aligners group [5]. Scott *et al.* however, did not discover any distinction between the self-ligating brackets and traditional fixed appliance brackets in terms of pain perception [6]. Nowadays clear aligners are gaining recognition among patients. Invisalign® was introduced to reduce discomfort to patient which was seen with conventional fixed orthodontic bracket procedure. Clear aligners were initially introduced to resolve mild to moderate dental crowding and close mild spacing. Clear aligners are transparent, thin plastic appliances formed using CAD computer aided manufacturing techniques, resembling splints covering teeth and gum margins. They are worn sequentially, moving teeth about 0.25-0.3 mm every 2 weeks [7]. With Invisalign®, teeth are moved gradually using a series of detachable clear polyurethane trays, or aligners. During orthodontic treatment, Invisalign® aligners (Align Technology, Santa Clara, CA, USA) provide better dental hygiene, less pain and increased aesthetics [8]. The shape memory polymers utilised in the removable aligners are cutting-edge, modern materials that can change their shape in response to outside stimuli while still being able to return to their initial configurations. The usage of clear detachable aligners has become very popular because to the current surge in demand for cosmetic orthodontic products [5]. There are limited studies existing on the pain perception of patients treated with Invisalign, passive self-ligating fixed appliances and conventional appliance. Therefore, it is of interest to assess the pain perception with Invisalign, passive self-ligating fixed appliances and conventional appliance at different time interval.

## Materials and Methods:

This prospective study was done in department of Orthodontics after obtaining approval from institutional ethics committee and consent from participants. The sample size was estimated by assuming 0.04 alpha, 0.30 beta and 80%. The participants were recruited from OPD department of Orthodontics. Participants were aged between 20-25 years of both genders. Total 75 participants were divided equally into 3 groups as; Group I- Invisalign, Group II-self ligating fixed appliances and Group III- conventional fixed appliance treatment. Inclusion criteria were; patients willing to participate with good oral health, no previous orthodontic treatment, no mucosal and periodontal diseases, no missing teeth, class I molar relationship indicated for non-extraction orthodontic treatment. The exclusion criteria was; dental students, patients on psychotropic drugs and who exhibited dental anxiety. All the treatment groups were in the initial alignment stage of orthodontic treatment. In all groups, the patients had minimal to moderate crowding, which was assessed using LII (range, 3-5). The study was done by trained investigator. The patients' experience of pain was assessed using a visual analogue scale (VAS) at six, twenty-four, forty-eight and one week following the appliance fitting. The questionnaire was closed-ended and coded. A 10-cm horizontal line was used for the VAS scoring, with "no pain" at the left end (score 0) and "very severe pain" at the right end (score 100). The study's evaluator was blinded in order to reduce bias in pain measurement. Questions concerning to frequency of analgesics, if any, used to treat pain were also answered by the participants. The obtained data was tabulated and statistically evaluated using SPSS statistical software version 22.0 using ANOVA test and Mann-Whitney U-test.

## Results:

The average age of the participants was 22±3.0 years. The study included 21men (42%) and 29 women (58%). Compared to fixed self-ligating and traditional fixed appliances, fewer patients treated with Invisalign aligners experienced discomfort, as seen in Table 1, which shows the presence of pain among the groups at various intervals. The variation was statistically considerable (P=0.001). There was drastically decline in number of participants experienced pain in Invisalign compared to fixed therapy over a time. Table 2 showed that, at 6 hours, 24 hours, 48 hours and 1 week, patients treated with Invisalign aligners experienced noticeably less discomfort (mean VAS ratings) than Group II and III. At 24 hours following the initial insertion of orthodontic equipment, the mean VAS ratings for Group I, Group II and Group III were 1.26, 5.98 and 3.64, respectively, indicating the highest degrees of pain in all patient groups. The difference between both groups is statically considerable (P=0.001). Pain perception was highest among conventional group compared to self-ligation group and least with Invisalign groups.

## Discussion:

Orthodontic treatment frequently involves pain complaints [9]. Because it enables the use of parametric tests, the visual analogue scale (VAS) is the most often used tool in scientific research [10]. During the initial orthodontic treatment, patients treated with Invisalign aligners, self-ligating brackets and traditional fixed brackets showed varying degrees of pain and discomfort. Compared to active and traditional self-ligating brackets, passive self-ligating brackets have less frictional resistance. As a result, it is believed that self-ligating brackets are less painful than traditional brackets [11]. The current study discovered that compared to patients treated with Invisalign aligners, a higher percentage of patients treated with passive self-ligating and traditional fixed appliances felt pain. Almasoud examined how patients who received Invisalign aligners and those who received passive self-ligating fixed appliances perceived pain. They came to the conclusion that Invisalign aligner users experienced less pain than those who used passive self-ligating fixed appliances [11]. These results are related to our findings. The degree of orthodontic pain following the placement of various orthodontic appliances has been compared in earlier research. When Bondemark *et al.* compared the amount of discomfort experienced when using spring and elastomeric separators, they found that the most severe pain occurred on day two and went away by day five [12]. According to Miller *et al.* during the first week of orthodontic therapy, patients reported less pain when wearing Invisalign aligners as opposed to fixed appliances [13]. The majority of patients in the current study reported pain 24 hours following the installation of the traditional fixed appliance and passive self-ligating device. These results contradict those of Shalish *et al.* who discovered that the majority of participants in the Invisalign group experienced pain on the first day [8]. Fujiyama *et al.* assessed the pain perception among Invisalign aligners and conventional fixed orthodontic appliances. They stated that, duration and intensity of pain during the initial stages of treatment were comparatively lower among patients treated with Invisalign aligners than edgewise orthodontic appliance [14]. Cardoso *et al.* from systemic review concluded that, pain was lesser with Invisalign compared to fixed appliance treatment [9]. Alturki *et al.* found higher pain intensity with fixed appliance compared to clear aligners [5]. Chan *et al.* found similar intensity of pain with clear aligner and fixed appliance [15]. According to Fujiyama *et al.* Invisalign might be less painful than the edgewise appliance [14]. The results of this study showed that patients treated with Invisalign aligners reported far less pain than those treated with self-ligating and traditional fixed appliances, which is consistent with the findings of earlier research. The study findings highlight the advantages of Invisalign in reducing pain perception in orthodontic patients. The drawback of the current study was smaller sample size. Further researches are needed on larger sample size to validate the findings.

## Conclusion:

Invisalign treatment group show lesser pain perception compared to that of fixed conventional and self-ligating groups. Clear aligners are patient friendly easy to use and maintain.

## Figures and Tables

**Table 1:** Percentage of participants experienced pain at different time interval in both groups

**Duration for pain assessment**	**Group I (Invisalign Aligners) (n-25)**	**Group II (self-ligating fixed appliances) (n-25)**	**Group III (Conventional fixed appliances) (n-25)**	**p- value**
6 hours	14 (56%)	24 (96%)		
24 hours	8 (32%)	21(84%)		0.001
48 hours	5 (20%)	18 (72%)		
1 week	1 (4%)	10 (40%)		

**Table 2:** Mean VAS scores at different time interval in both groups

**Duration for pain assessment**	**Group I (Invisalign Aligners)**	**Group II (self-ligating fixed appliances) (n-25)**	**Group III (Conventional fixed appliances) (n-25)**	**p- value**
6 hours	1.18 ± 1.64	5.36 ± 3.14	6.36 ± 3.14	
24 hours	1.26 ± 2.58	5.98 ± 3.32	6.12 ± 3.32	0.001
48 hours	0.58 ± 1.73	4.43 ± 3.23	5.73 ± 3.23	
1 week	0.37 ± 1.56	2.54 ± 2.53	3.64 ± 2.53	
